# Expression of extracellular matrix protein in advanced keratoconus and the normal human cornea

**DOI:** 10.3389/fmed.2025.1612452

**Published:** 2025-08-05

**Authors:** Fatima Habroosh, Perla Filippini, Ahmed Al Saadi, Shady Soliman, Burcin Kepez Yildiz, Prity Sahay, Dalia G. Said, Harminder S. Dua

**Affiliations:** ^1^Academic Ophthalmology, Division of Clinical Neuroscience, School of Medicine, University of Nottingham, Nottingham, United Kingdom; ^2^Department of Ophthalmology, Sheikh Khalifa Medical City, Abu Dhabi, United Arab Emirates; ^3^Research Institute of Ophthalmology, Cairo, Egypt; ^4^Department of Ophthalmology, University of Health Sciences Cam and Sakura City Hospital, Istanbul, Türkiye

**Keywords:** keratoconus, descemet membrane, elastin, keratoplasty, elasticity

## Abstract

**Background:**

The pre-Descemet layer (Dua’s layer) (PDL/DL) and the Descemet membrane (DM) have the highest expression of elastin in normal corneas. Reduced expression of elastin occurs in the corneal stroma of patients with keratoconus (KC). This study aimed to examine the expression of elastin and that of other extracellular matrix (ECM) components, and proteoglycan proteins in the PDL/DL and DM in advanced KC.

**Methods:**

Corneal tissue obtained from four subjects with KC who underwent penetrating keratoplasty was compared with tissue from four normal corneas. The tissues were sectioned and analyzed with specific markers for extracellular matrix proteins and proteoglycans—namely, elastin, collagens I, IV, and VI, fibulin-2, and decorin—using immunohistochemical analysis. Fluorescence intensity was measured using ImageJ software and compared to controls.

**Results:**

The expression of elastin was significantly reduced in the PDL/DL and DM in KC cases compared to controls. Concurrently, an increased expression of elastin was noted anteriorly in the subepithelial region. Expression of collagen IV, collagen VI, and decorin was reduced in the PDL/DL and DM, whereas expression of collagen I and fibulin-2 was unaltered in KC cases compared to controls.

**Conclusion:**

The significant reduction of elastin in the PDL/DL and DM in cases of advanced keratoconus with acute corneal hydrops (ACH) may play a crucial role in the pathology of KC and the subsequent onset of ACH. Furthermore, elastin is considered to be pivotal in the disease progression, as keratoconus typically starts with a posterior elevation affecting these two layers.

## Introduction

Keratoconus (KC) is a relatively common progressive ectatic disorder of the cornea, affecting approximately 1 in 375 individuals in the general population (1). It asymmetrically affects young individuals, with one eye often worse than the other. KC can cause considerable visual impairment that can be addressed through the use of spectacles, contact lenses, and a range of laser and surgical interventions. As it is a progressive condition, the affected individuals often go through a wide range of treatment options (2). One significant treatment is collagen cross-linking, wherein the anterior part of the cornea is strengthened using ultraviolet light and riboflavin. This method has proven effective in arresting or slowing the progression of KC (3, 4).

The wide array of treatment options reflects the range of mechanisms involved in the etiopathogenesis of KC. Genetic factors, inflammation, and biochemical changes, including alterations in extracellular matrix (ECM) composition and enzymatic degradation of the cornea, are all implicated in KC. Mechanical factors, with eye rubbing being the most consistent, also play a significant role in the condition (5, 6). As early KC often begins in the posterior cornea with posterior elevation and thinning, attention has focused on this part of the cornea, particularly the posterior stroma, the pre-Descemet layer (Dua’s layer) (PDL/DL), and the Descemet membrane (DM) (2, 7, 8). This area is also significantly affected in acute corneal hydrops (ACH), which is a dramatic event in the natural course of KC in some patients (9, 10). To further understand the pathogenesis of KC, efforts are focused on determining the extracellular matrix (ECM) remodeling in the tissues involved. Dysregulation in the formation and assembly of ECM proteins, proteoglycans, and glycosaminoglycans is implicated in the pathogenesis of KC. Unique structural properties of the DM and stroma have also been reported, which may have implications for lamellar corneal transplantation (11, 12). A decrease in collagen types I and VI was observed in the KC epithelium, whereas collagen types III, IV, and V were found to be lower in the KC stroma. This indicates a loss of tensile strength in the cornea, which could be a factor in the progression of ectasia (13, 14). To understand whether differences in structural and extracellular proteins between normal and KC corneas could play a role in the etiopathogenesis of KC and ACH, we examined the presence and distribution of proteoglycans and ECM proteins—namely elastin, collagens I, IV, and VI, fibulin-2, and decorin—using immunohistochemical analysis in KC cases compared to normal corneas. Our results indicate the preferential involvement of the posterior layers in the etiopathogenesis of KC.

## Methods

### Sample preparation

Four human corneal buttons obtained from patients with KC who underwent penetrating keratoplasty following an episode of ACH (3 corneas) or advanced cone (1 cornea) were examined. The average age of the patients was 35 years. After the surgery, the corneal buttons were stored in Eagle’s minimum essential medium (MEM, ThermoFisher Scientific, United Kingdom). Similarly, four control normal corneas stored in Eagle’s MEM were obtained from the UK National Health Service Blood and Tissue Bank. The mean donor age was 70 years. The KC case and normal control corneas were cut into small pieces, approximately 0.5 cm in size, under a dissection microscope (Leica S6 D, Wetzlar, Germany). The tissue specimens were washed thoroughly with sterile 1X phosphate-buffered saline (PBS). Then, the tissues were fixed in 4% paraformaldehyde (PFA) at 4°C for 2 h, washed with 1X PBS to remove the excess fixing reagent, and incubated overnight at 4°C in a 30% sucrose solution on a rotating mixer. Then, the tissue samples were embedded in an optimal cutting temperature (OCT) compound, a cryoembedding matrix, and stored at −80°C before sectioning. Ethical approval was obtained from the local ethics committee, and this study adhered to the principles of the Declaration of Helsinki (Amendment number: 06147 SA09).

### Immunofluorescence (IF)

ECM proteins such as elastin and collagens I, IV, and VI, along with proteoglycan and glycosaminoglycan proteins such as fibulin-2 and decorin, were evaluated using the IF assay. Furthermore, 10 μm cryostat (Leica, Wetzlar, Germany) sections were permeabilized in permeabilization buffer (0.05% Triton X-100 in 1X PBS) for 15 min. The sections were then blocked with 10% normal goat/donkey serum for 1 h at room temperature (RT) and were sequentially incubated overnight with specific primary monoclonal or polyclonal antibodies at appropriate dilutions (Table 1) in antibody dilution buffer (5% normal goat/donkey serum in permeabilization buffer). Each section was washed thrice with 1X PBS for 5 min per wash. Following this, the sections were incubated with the respective fluorescent dye-conjugated secondary antibodies at appropriate dilutions (Table 1) in antibody dilution buffer at RT for 1 h in the dark. The sections were again washed three times with 1X PBS, and mounted with mounting medium (ProLong Diamond Antifade Mountant, Invitrogen, USA) and a coverslip. The sections were imaged using a fluorescence microscope (Leica Microsystems CMS GmbH; Model DMIL LED Fluo; Wetzlar, Germany). The images were analyzed using Leica Application Suite X (version 3.0.0.15697), and the composite images were prepared using Adobe Photoshop CS5 (Adobe Systems Inc., San Jose, CA). Immunostaining with isotype controls—namely, rabbit isotype monoclonal control and rabbit isotype polyclonal control antibodies against collagen I, collagen VI, elastin, and fibulin-2—was performed to exclude false-positive results related to cross-reactivity.

**Table 1:** List of primary and secondary antibodies used in this study.

Antibodies	Catalog number	Source	Clonality	Dilutions	Supplier
Collagen I	ab34710	rabbit	P	1:200	Abcam
Collagen IV	AB769	goat	P	1:25	Merck Millipore
Collagen VI	ab182744	rabbit	M	1:50	Abcam
Elastin	ab217356	rabbit	P	1:50	Abcam
Decorin	AF143	goat	P	1:25	R&D System
Fibulin-2	ab234993	rabbit	P	1:100	Abcam
Alexa flour™ 488 anti-rabbit IgG	A21206	donkey	P	1:200	Fisher Scientific
Alexa flour™ 488 anti-goat IgG	A11008	donkey	P	1:200	Fisher Scientific
Alexa flour™ 594 anti-goat IgG	A11058	donkey	P	1:200	Fisher Scientific

M, monoclonal; P, polyclonal.

### Images and statistical analysis

Images were captured using a fluorescence microscope at 10X and 20X magnifications. The images were analyzed for fluorescence intensity of PDL/DL and the DM (corrected total cell fluorescence = Integrated Density – (Area of selected cell X Mean fluorescence of background readings)) using ImageJ software (National Institutes of Health, Bethesda, Maryland, USA).

GraphPad Prism 10.0.2 was used to plot a graph to compare the fluorescence intensity of each protein analyzed using a grouped comparison. The results are presented as the mean ± standard deviation (SD) for each KC patient and control sample. A two-way ANOVA with Šídák’s multiple comparisons test was performed to calculate statistical significance, with a *p*-value of <0.05.

## Results

**Figure 1:**
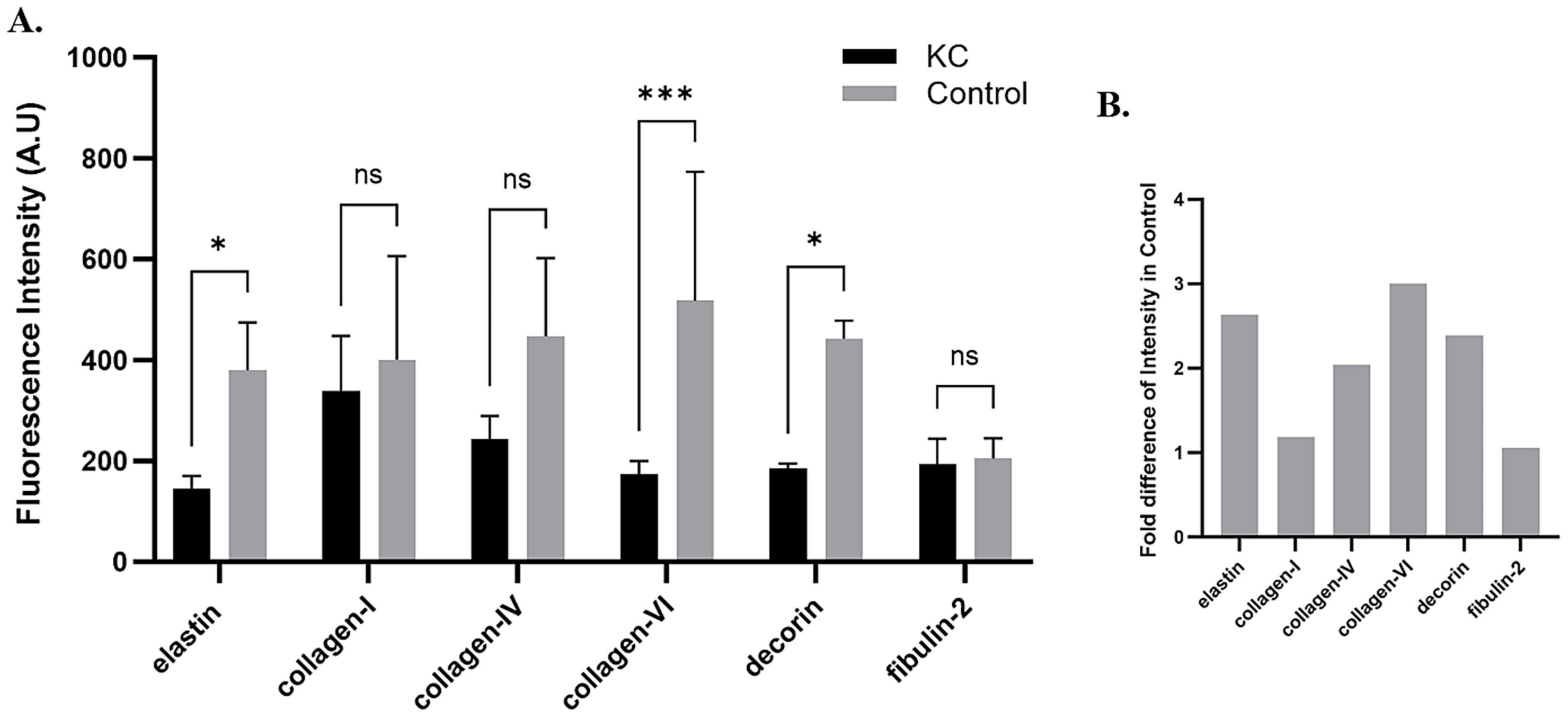
Fluorescence intensity of each protein in keratoconus (KC) corneas and normal controls. **(A)** A bar graph showing the fluorescence intensity of each protein studied in the pre-Descemet layer (Dua’s layer) (PDL/DL) and the Descemet membrane (DM) in the keratoconus corneas and normal control corneas. Image J software was used to analyze the intensity of fluorescence in each tissue. The corrected total cell fluorescence intensity was calculated by normalizing the selected area of fluorescence intensity with the background reading. **(B)** A graph showing the fold change in fluorescence intensity normalized with the KC’s cases intensity readings to the control’s intensity readings of each protein. Means ± SD shown, *p < 0.05, ***p < 0.001, two-way ANOVA with Šídák’s multiple comparisons test. KC, Keratoconus; ns, not significant.

**Figure 2:**
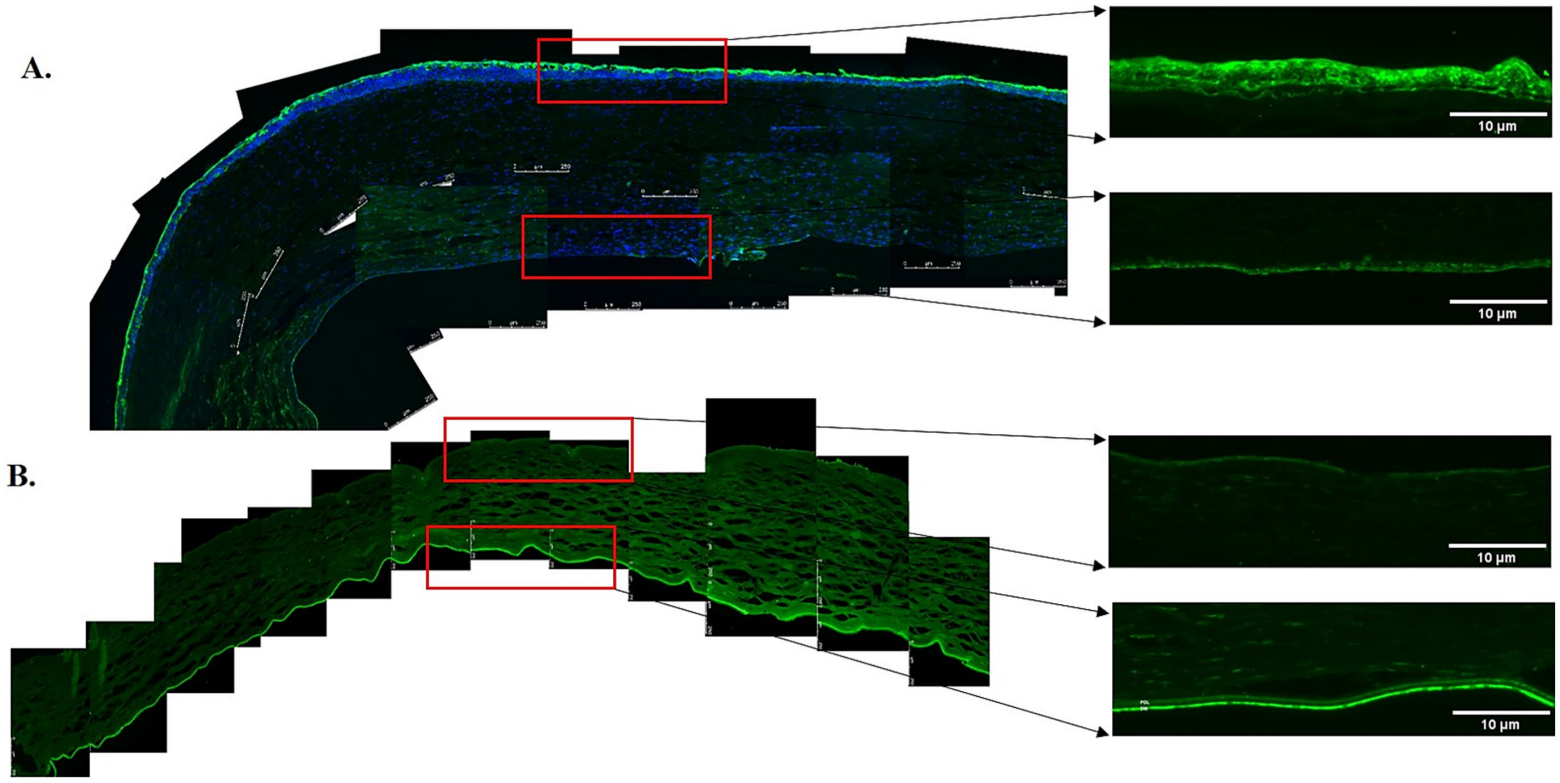
Immunofluorescent staining of elastin in the anterior and posterior corneas of the keratoconus (KC) cases and controls. **(A)** A representative stitched image of the KC posterior cornea at 10x magnification (left and lower panels) and at 20x magnification (right and lower panels) showing the reduced intensity of elastin (green) in the pre-Descemet layer (Dua’s layer) (PDL/DL) and the Descemet membrane (DM). Increased intensity of elastin (green) at 10x magnification (left and upper panels) and 20x magnification (right and upper panels) was observed in the sub-epithelial region of the anterior cornea, corresponding to the Bowman’s zone. **(B)** The control corneas showed high-intensity elastin (green) staining in the PDL/DL and DM layers at 10x magnification (left and lower panels) and at 20x magnification (right and lower panels), with no fluorescent staining for elastin (green) at 10x magnification (left and upper panels) and 20x magnification (right and upper panels) in the sub-epithelial region of the anterior cornea. Higher magnification (20x) images were highlighted with a red rectangle for the PDL/DL and DM of the posterior cornea (right and lower panels) and the sub-epithelial region of the anterior cornea (right and upper panels). Magnifications and scale bars: 10x (scale bar = 250 μM) and 20x (scale bar = 10 μM).

**Figure 3:**
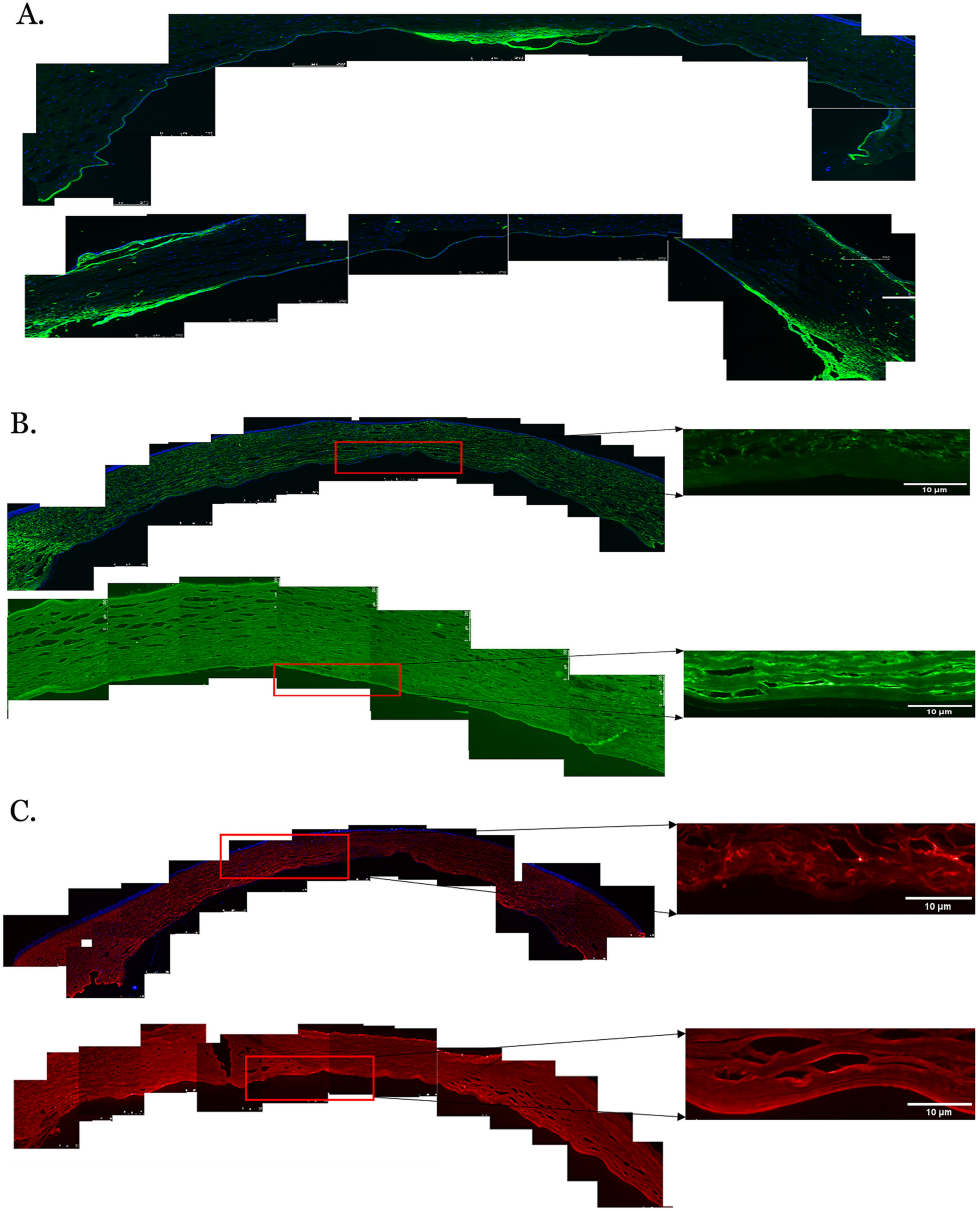
Immunofluorescent staining of proteins in the posterior corneas of keratoconus (KC) cases and controls. **(A)** A representative stitched image of the posterior cornea showing that the distribution of collagen IV (green) was not uniform in the KC cases (upper panel) and control corneas (lower panel). Decreased fluorescence intensity was observed in the KC cases compared to the normal controls. **(B)** The fluorescent intensity of collagen VI (green) was considerably reduced in the posterior stroma, the pre-Descemet layer (Dua’s layer) (PDL/DL), and the Descemet membrane (DM) in the KC cases at 10x magnification (left and upper panels) and 20x magnification (right and upper panels) compared to the normal controls at 10x magnification (left and lower panels) and 20x magnification (right and lower panels). **(C)** The fluorescent intensity of decorin (red) in the posterior cornea was considerably reduced in the posterior stroma, PDL/DL, and DM in the KC cases at 10x magnification (left and upper panels) and 20x magnification (right and upper panels) compared to the normal controls at 10x magnification (left and lower panels) and 20x magnification (right and lower panels). Higher magnification (20X) images were highlighted with a red rectangle for the PDL/DL and DM (right panel). Magnifications and scale bars: 10x (scale bars = 250 μM) and 20x (scale bars = 10 μM).

Elastin expression in the posterior cornea (PDL and DM) of control corneas was statistically significantly higher by a magnitude of 2.6-fold (*p* = 0.0404), compared to KC cases. The distribution was homogeneous throughout the posterior layers of the control cornea. In contrast, elastin staining intensity was increased in the subepithelial layers of the KC eyes compared to the normal corneas (Figures 1, 2). The distribution of collagen IV was not uniform in the posterior part of the cornea in both KC cases and control corneas. Its expression was reduced by a magnitude of 2.0-fold in KC cases compared to control corneas (Figures 1, 3), although this difference was not statistically significant. Collagen VI was present uniformly in all corneal layers, including the posterior part. In KC corneas, there was a three-fold reduction in fluorescence intensity compared to control corneas (*p* = 0.0010) in the posterior cornea (Figures 1, 3). Decorin expression was uniformly reduced throughout the whole cornea, especially in the posterior cornea. Moreover, the expression of decorin was significantly reduced by 2.4-fold (*p* = 0.0206) in KC cases compared to control corneas (Figures 1, 3). However, the expression of collagen I and fibulin-2 showed an insignificant reduction with a fold change of 1.0 (Figure 1) in fluorescence intensity in KC cases compared to normal corneas (Supplementary Figure S1). The isotype controls did not show any positive staining in the tissue specimens (Supplementary Figure S2), indicating that there were no cross-reactivity-related positive results.

## Discussion

KC is a multifactorial disease with a complex etiopathogenesis that leads to biomechanical alterations of the cornea. As the cornea becomes thin and ectatic, it causes distortion in light transmission and focusing, resulting in significant visual impairment (2). Corneal tomography, which offers information about the posterior and anterior surfaces of the cornea, provides evidence that early KC begins in the posterior cornea in the form of thinning and elevation of the posterior corneal plane (7). Hence, some clues regarding the etiopathogenesis of KC may lie in the posterior cornea, including the endothelial cells, the DM, the PDL/DL, and the posterior stroma. Recent evidence suggests that ACH, a dramatic occurrence in the natural history of KC in some cases, is caused by the rupture of the DM and PDL/DL (6, 9, 15, 16). This further emphasizes the importance of the posterior cornea in the pathogenesis of KC.

Several molecules have been implicated in the pathogenesis of KC. These molecules may show altered, increased, or reduced expression and are associated with structural and biochemical changes. For example, these biochemical changes include an increased expression of MMP-1 and MMP-9, reduced levels of enzymes such as superoxide dismutase, catalase, and glutathione peroxidase, suggesting oxidative stress, and an upregulation of several proinflammatory cytokines, including IL-6 and TNFα (5). This overview is just a snapshot of the changes in KC, but the important point is that these findings relate to the entire corneal stroma and have not been reported as specific to different stromal locations or layers.

One molecule, elastin, is present as a uniform network throughout the corneal stroma (17); however, en-face serial scanning electron microscopy revealed that it is specifically distributed as an annulus along the limbus and is highly concentrated in the PDL/DL (10, 18). This was further substantiated and localized by IF staining, which demonstrated elastin presence throughout the entire thickness of the PDL/DL and as a narrow band in the anterior part of the DM, most likely corresponding to the banded layer (19). It has been suggested that the high elastin content in the PDL/DL confers considerable elasticity to this layer, as observed during deep anterior lamellar keratoplasty (DALK) with a type 1 big bubble (20). The elastin content and distribution in the DM have been shown to confer elasticity and give it its unique characteristic of scrolling, with the endothelial cells on the outside of the scroll. Treatment of the DM scroll with elastase resulted in complete unscrolling of the DM and corresponding degradation of the anterior elastin band (21).

Using serial block-face scanning electron microscopy and transmission electron microscopy (10), it was found that the elastic fiber distribution was deficient in the PDL/DL of keratoconic corneas compared to normal corneas. Previous studies have focused only on the stroma anterior to the DM (i.e., the PDL/DL) and not on the elastic fiber content of the DM. In our study, we were able to demonstrate the lack of elastin staining in both the PDL/DL and the DM, where it is normally present in abundance, as seen in our control eyes. This lack of elastin in the PDL/DL and DM could alter the biomechanics of these tissues and contribute to the posterior changes seen in KC. The DM and PDL/DL may bow forward but not return to the original positions due to the lack of elastin. Later in the course of the disease, further loss or absence of elastin could render these tissues susceptible to rupture upon stretching, as observed in ACH. Interestingly, White et al. (10) noted that there was increased elastic fiber density under the epithelium in the anterior cornea, which was exactly what we found with our immunostaining techniques. This evidence adds another dimension to the pathogenesis of KC that needs to be explored further.

Dua et al. (6, 15, 19) provided early evidence for a break in both the PDL/DL and DM in the pathogenesis of ACH. While performing a Bowman’s layer transplant, Parker et al. noted that inadvertent perforation of the PDL/DL and DM during the creation of the lamellar pocket resulted in ACH, which did not occur when the procedure was associated with a DM tear alone (16). This provided direct evidence that rupture of the PDL/DM is essential in the pathogenesis of ACH. It was further shown that incisions to the PDL/DL and DM in normal corneas did not result in ACH, leading to the conclusion that the abnormal collagen and ground substance content in KC corneas is also required for the development of ACH (9, 15, 19). Furthermore, it has been shown that the rapid resolution of ACH can occur after the approximation of the torn edges of the PDL/DL with mattress sutures, even without approximating the torn edges of the DM (20).

Despite the significant advantage of retention of host endothelial cells offered by deep anterior lamellar keratoplasty over penetrating keratoplasty in KC treatment, the tissue samples available for our study and that of White et al. (10) were obtained from patients with advanced KC requiring a full-thickness graft. The majority of our patients had a history of ACH. Therefore, the results are biased toward advanced KC. Direct evidence of elastin loss in early KC is not available, but it can be inferred from the occurrence of posterior elevation in early KC.

Collagen VI has been implicated in the pathogenesis of KC. Vogt’s striae in the posterior cornea of KC patients are predominantly composed of collagen VI fibrils. KC patients with Vogt’s striae are at a higher risk of developing ACH compared to KC patients without Vogt’s striae (5). Our study showed a significant reduction in collagen VI in the posterior cornea in KC, which is in alignment with the above-mentioned study. Similarly, the expression of other molecules in stromal glycosaminoglycans and proteoglycans—including lumican, keratocan, biglycan, and decorin—decreases significantly throughout the corneal stroma in KC (5). We demonstrated that decorin was specifically reduced in the posterior cornea. Our data indicate that elastin is significantly reduced in KC, and other molecules that are generally reduced throughout the whole corneal stroma in KC, namely collagen VI and decorin, are also significantly reduced in the PDL/DL. These differential changes in the posterior cornea compared to the anterior cornea could provide insights into why early KC commences in the posterior cornea and why, in advanced KC, the dramatic event of ACH occurs due to the rupture of these layers.

The findings from this study add to the body of knowledge on the changes that occur in KC and may explain events such as ACH. However, these results should be interpreted in the context of the limitations posed by the small number of patients and the disparity in age ranges between the KC tissue samples and control samples.

The majority of patients with advanced KC requiring corneal grafting undergo deep anterior lamellar keratoplasty (DALK). In this procedure, the PDL and the DM are not removed and are therefore unavailable for analysis. Only patients undergoing full-thickness/penetrating keratoplasty graft (PK) have these layers in the tissue that is removed from the patient. Therefore, it is very difficult to obtain host tissue samples in large numbers. Moreover, patients who have experienced acute corneal hydrops (ACH) are not only poor candidates for DALK but also have distortion or scarring of the cornea in the visual axis. As a result, penetrating keratoplasty (PK) is frequently the preferred surgical option. Consequently, the tissues in our study were obtained from advanced KC patients with acute hydrops. For the same reasons, it is very difficult to obtain tissues with preserved PDL/DM from younger patients with KC who have not experienced ACH, as for them, DALK, not PK, is the procedure of choice. Nevertheless, despite the small sample size, the findings reported are novel and of significance for the corneal transplant community. We have also presented data (Supplementary Figure S3) demonstrating that the elastin content in the DM of young donor tissue was similar to that in adults (controls), but elastin levels in the DM of the young eyes with ACH were significantly reduced. A minimum of three samples (*n* = 3) is typically required to apply basic statistical tests, and in our study, we had four samples. Although this is a small sample size, the results are still valid.

Future studies aimed at examining the elastin content of the PDL and DM across different age groups, both young and old, and the comparative elasticity of the respective tissues will help us understand in more detail the relationship between elastin content and elasticity, and their roles in DM/PDL rupture and the development of ACH.

## Data Availability

The original contributions presented in the study are included in the article/Supplementary material, further inquiries can be directed to the corresponding author.

## Supplementary material

SUPPLEMENTARY FIGURE S1 Immunofluorescence staining of proteins in the keratoconus (KC) cases and control corneas. **(A)** The distribution of the fluorescence intensity for collagen I (green) did not alter in KC cases (upper panel) as compared to control corneas (lower panel). **(B)** The distribution of the fluorescence intensity for fibulin-2 (green) did not alter in KC cases (upper panel) as compared to control corneas (lower panel).

SUPPLEMENTARY FIGURE S2 The fluorescence intensity of isotype control. The fluorescence intensity of the isotype control of polyclonal (upper panel) and monoclonal (lower panel) is low.

SUPPLEMENTARY FIGURE S3 The immunofluorescence staining of elastin protein and its expression in Descemet membrane (DM) at 10x magnification of young cornea donor at ages 34 **(A)**, 39 **(B)** and 45 **(C)**.
