# Supplementary material for: Expression of extracellular matrix protein in advanced keratoconus and the normal human cornea
Source: Front Med (Lausanne). 2025 Aug 5;12:1612452. doi: 10.3389/fmed.2025.1612452 (PMC12361212; doi:10.3389/fmed.2025.1612452)

**Supplementary Material**

**Supplementary Figure S1.**


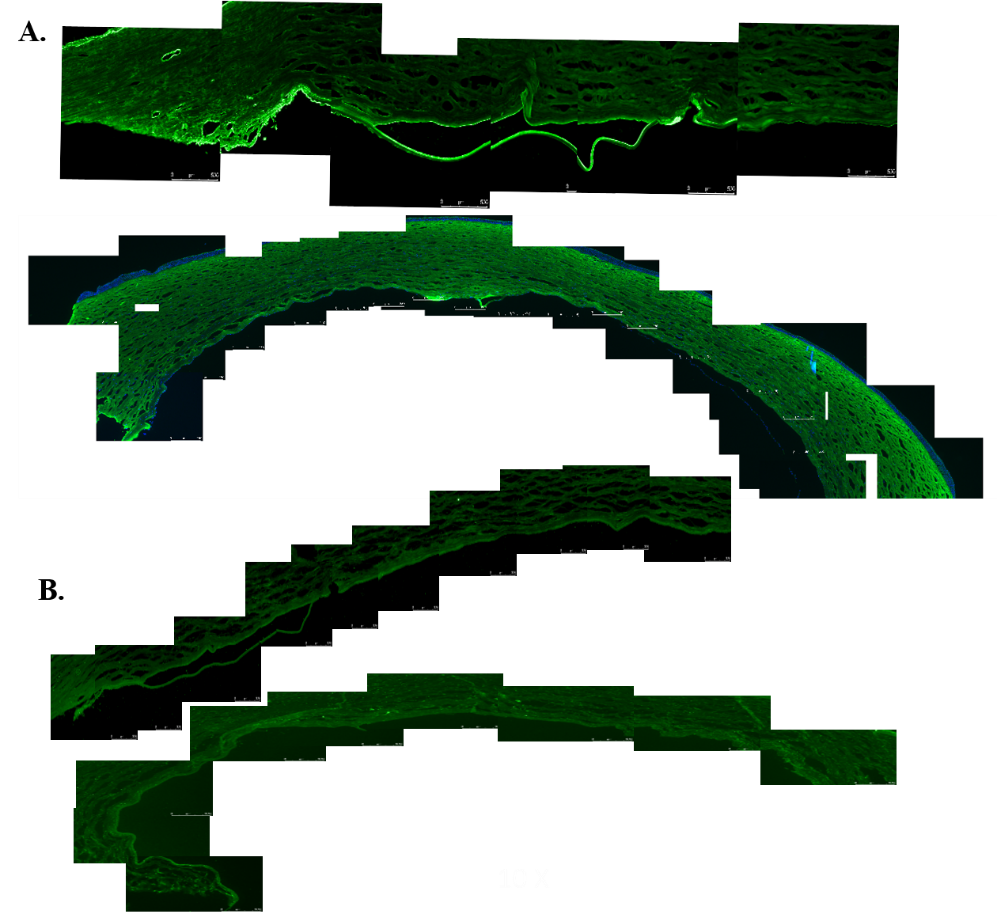


**Supplementary Figure S2.**


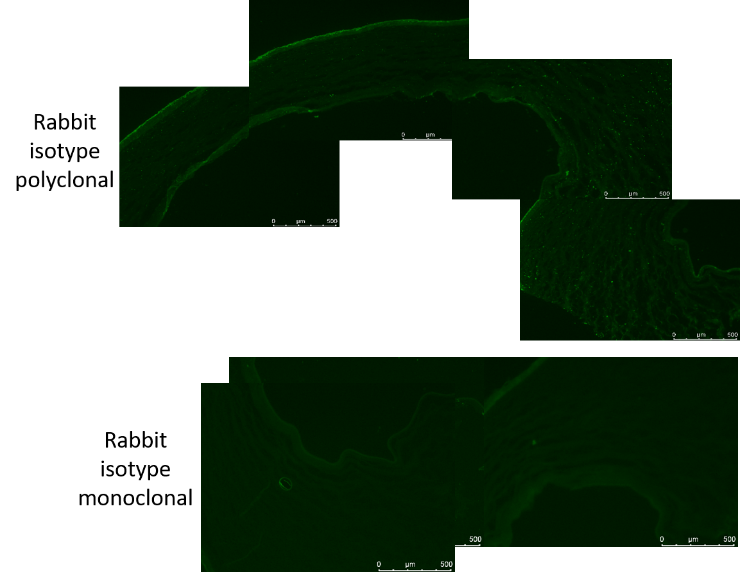


**Supplementary Figure S3.**


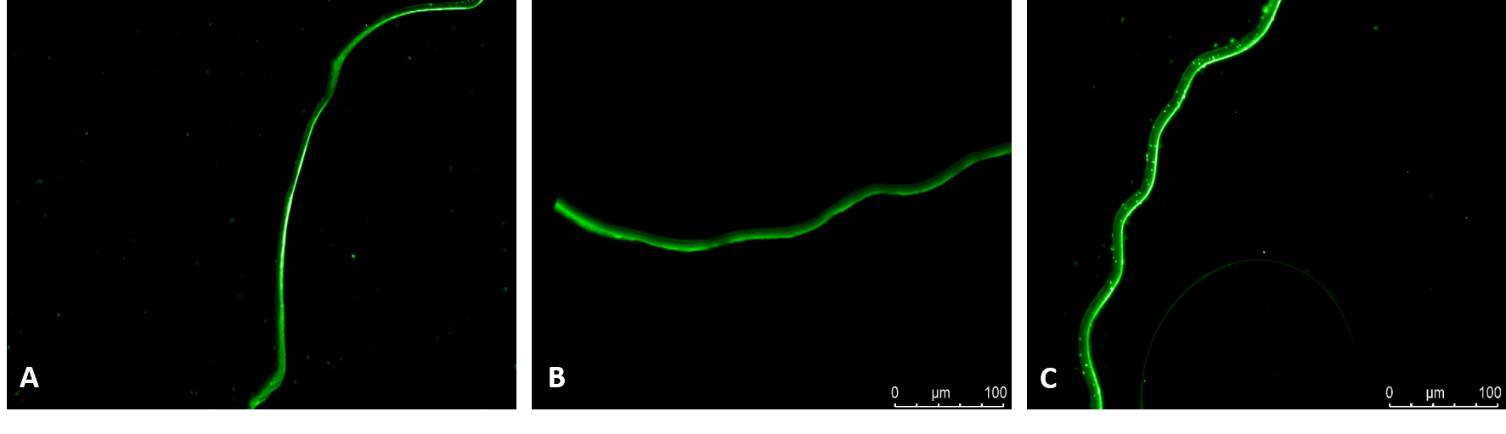

Supplement: SUPPLEMENTARY FIGURE S1 — Immunofluorescence staining of proteins in the keratoconus (KC) cases and control corneas. (A) The distribution of the fluorescence intensity for collagen I (green) did not alter in KC cases (upper panel) as compared to control corneas (lower panel). (B) The distribution of the fluorescence intensity for fibulin-2 (green) did not alter in KC cases (upper panel) as compared to control corneas (lower panel). [file Data_Sheet_1.docx]
